# The Association of Fatigue With Decreasing Regularity of Locomotion During an Incremental Test in Trained and Untrained Healthy Adults

**DOI:** 10.3389/fbioe.2021.724791

**Published:** 2021-11-24

**Authors:** Marco Rabuffetti, Mathias Steinach, Julia Lichti, Hanns-Christian Gunga, Björn Balcerek, Philipp Nils Becker, Michael Fähling, Giampiero Merati, Martina Anna Maggioni

**Affiliations:** ^1^ IRCCS Fondazione Don Carlo Gnocchi, Milano, Italy; ^2^ Charité - Universitätsmedizin Berlin, corporate member of Freie Universität Berlin, Humboldt-Universität zu Berlin, Institute of Physiology, Center for Space Medicine and Extreme Environments Berlin, Berlin, Germany; ^3^ Charité - Universitätsmedizin Berlin, corporate member of Freie Universität Berlin, Humboldt-Universität zu Berlin, Institute of Vegetative Physiology, Berlin, Germany; ^4^ Department of Biotechnology and Life Sciences (DBSV), University of Insubria, Varese, Italy; ^5^ Department of Biomedical Sciences for Health, Università degli Studi di Milano, Milano, Italy

**Keywords:** healthy subjects, locomotion, regularity, fatigue, Bruce test, training

## Abstract

Fatigue is a key factor that affects human motion and modulates physiology, biochemistry, and performance. Prolonged cyclic human movements (locomotion primarily) are characterized by a regular pattern, and this extended activity can induce fatigue. However, the relationship between fatigue and regularity has not yet been extensively studied. Wearable sensor methodologies can be used to monitor regularity during standardized treadmill tests (e.g., the widely used Bruce test) and to verify the effects of fatigue on locomotion regularity. Our study on 50 healthy adults [27 males and 23 females; <40 years; five dropouts; and 22 trained (T) and 23 untrained (U) subjects] showed how locomotion regularity follows a parabolic profile during the incremental test, without exception. At the beginning of the trial, increased walking speed in the absence of fatigue is associated with increased regularity (regularity index, RI, a. u., null/unity value for aperiodic/periodic patterns) up until a peak value (RI = 0.909 after 13.8 min for T and RI = 0.915 after 13.4 min for U subjects; median values, n. s.) and which is then generally followed (after 2.8 and 2.5 min, respectively, for T/U, n. s.) by the walk-to-run transition (at 12.1 min for both T and U, n. s.). Regularity then decreases with increased speed/slope/fatigue. The effect of being trained was associated with significantly higher initial regularity [0.845 (T) vs 0.810 (U), *p* < 0.05 corrected], longer test endurance [23.0 min (T) vs 18.6 min (U)], and prolonged decay of locomotor regularity [8.6 min (T) vs 6.5 min (U)]. In conclusion, the monitoring of locomotion regularity can be applied to the Bruce test, resulting in a consistent time profile. There is evidence of a progressive decrease in regularity following the walk-to-run transition, and these features unveil significant differences among healthy trained and untrained adult subjects.

## Introduction

Locomotion is characterized by pseudo-periodic patterns of many kinematic and kinetic variables, and these are related to any anatomical limb and to the center of mass. Together, these movements are referred to “stride”, or the basic “gait cycle” (Bovi et al., 2011). Such pseudo-periodic patterns may display different gradations in regularity, ranging from the ideal cyclic pattern (i.e., mathematically, a sum of sinusoids according to the Fourier series approach and typical of many physical phenomena in a conservative field such as the pendulum oscillation) to patterns reflecting disruptions in gait regularity. While more regular locomotor patterns have been demonstrated to be related to behavioral factors [such as an increase in gait speed (Rabuffetti et al., 2019)], it has been shown that various alterations of the neuromuscular system may decrease locomotion regularity (Tura et al., 2010; Demonceau et al., 2015; Barden et al., 2016).

Quantification of locomotion regularity is a feature contributing to gait assessment and performance ability, possibly supporting the design and outcome appraisal of therapeutic and rehabilitative interventions (Auvinet et al., 2017). Factors that modulate regularity must therefore be identified and described to understand the underlying physiological mechanism. The objective assessment of locomotor regularity can be observed using any method of gait analysis. Wearable inertia sensors are the apparatus of choice due to the ease of use and the sensors’ low cost. It is noteworthy that regularity is not only limited to the variability of locomotion’s spatiotemporal parameters [an approach which prevails in literature (Hamacher et al., 2011)] but is also intended as the similarity of the time patterns of variables related to the movement in consecutive strides (Tura et al., 2010). Methods based on the autocorrelation analysis of accelerometric signals have become the *de-facto* standard to quantify the level of regularity of pseudo-periodic patterns of human walking and running (Auvinet et al., 2002; Moe-Nilssen and Helbostad, 2004).

Increasing locomotion speed is a functional factor, which has been demonstrated to be associated with increased locomotion regularity. Particularly, regularity increases with increased walking speed and reaches a plateau when switching to running (Rabuffetti et al., 2019). After switching to running, a further increase in speed does not produce additional regularity increases, since the regularity index values will have already come very close to the theoretical upper limit. The previous observation was limited to running at speeds close to the walk-to-run transition. In the cited experiments (Rabuffetti et al., 2019), walking and running at different velocities were analyzed in bouts lasting 60 s with recovery resting phases in between. These resting phases were instigated in order to avoid fatigue effect, which is expected in prolonged motor performances (Yoshino et al., 2004; Enoka and Duchateau, 2016). This naturally develops in more demanding locomotor tasks, such as in a well-established standard ramped treadmill test to assess cardiac function and fitness level executed with the Bruce protocol (Bruce et al., 1963, 2004). This test starts from a resting standing position and requires locomotion on a treadmill at progressively increasing speed and slope until the subject’s volitional exhaustion. Regularity is expected to be initially affected by speed changes and then later by fatigue in the last phase of the Bruce protocol. Furthermore, modulating training effects have also been observed in other physiological parameters (Balcerek et al., 2020). In terms of locomotor regularity, previous studies have shown an association between fitness level and gait variability (Ciprandi et al., 2018).

The use of this protocol in both trained and untrained healthy adults would allow for the identification of a possible effect of aerobic training on the fatigue–regularity association. It is well-documented that both central and peripheral fatigue exert a significant effect on isolated muscle performance, which differs between T and U individuals. However, the evaluation of fatigue effects in complex motor tasks (such as free walking and running) is more challenging. This is especially the case in the field where fatigue signs following team matches or running races cannot easily be characterized from a biomechanical and physiological point of view. Furthermore, besides training level, fatigue symptoms during competitions are largely influenced by other several factors such as technique, motor skills, motivation, the opponents’ behavior, etc. An interesting review of the interactive processes that may connect the multiple symptoms of fatigue during sports activities was published by Knicker et al. (2011).

Our working hypotheses are that the fatigue that develops in the final stages of the Bruce protocol will negatively affect locomotion regularity and that fitness level may affect locomotion regularity as well. These hypotheses are in line with previous literature regarding regularity (Schütte et al., 2015) and adopting different metrics based on a state space approach (Riazati et al., 2020) while comparing fatigue effects before and after exercise (Schütte et al., 2015; Riazati et al., 2020). Given that contrasting factors affect regularity in the Bruce protocol (i.e., positive for speed increments and negative for fatigue accumulation), a simple linear model is not expected to fit well. We therefore considered a non-linear model to track regularity during the Bruce test execution.

The present study aimed to 1) analyze the association between fatigue observed during the Bruce test and locomotion regularity assessed by an autocorrelation method and 2) evaluate the effect of the training level on the fatigue–regularity association during the Bruce test.

## Methods

### Subjects

Fifty healthy adult volunteers (27 males and 23 females) were enrolled as part of a larger study. Details related to recruitment and inclusion criteria are reported elsewhere (Balcerek et al., 2020). Upon volunteering, potential subjects were screened for inclusion criteria, received detailed information about the experiment, and were given the opportunity to ask questions and take their time to decide whether they wanted to participate. All volunteers gave their informed written consent. The study was approved by the Charité Ethics Committee (EA1/154/18). All procedures and measurements complied with the Declaration of Helsinki (54^th^ Revision 2008; Korea)^1^ regarding human subjects.

### Experimental Protocol

On the testing day, subjects were invited to the Charité Laboratory in Berlin in the morning (9 a.m.–12 p.m.). All tests were conducted between February and April 2019. First, volunteers received further details about the study. Afterwards, they underwent a detailed interview with the principal investigators describing their training experiences and their current regular exercise routines (or lack thereof). The experiment was carried out on a motorized treadmill (H/P/Cosmos, Pulsar, Nussdorf-Traunstein, Germany), and the subjects went through the Bruce protocol (Bruce et al., 1963, 2004) until their point of maximum fatigue/exhaustion was reached. Oxygen consumption and carbon dioxide production were continuously measured from expired air using a breath-by-breath gas analysis system (Metalyzer 3B, Cortex Biophysik GmbH, Leipzig, Germany) connected to a computer for data collection (Metasoft 3, version 3.9.9 SR1, Cortex Biophysik GmbH, Leipzig, Germany).

The Bruce protocol implements a ramped test where the velocity is gradually increased along with the slope. This is a well-established test to assess maximal oxygen consumption and is generally well tolerated by subjects, independent of their training level. However, to quantify the higher performances of endurance-trained subjects, two more stages were added to the original protocol, resulting in the protocol reported in Table 1. The subjects were free to make a walk-to-run transition and were allowed to quit at any point at which they felt exhausted and unable to safely continue. However, upon reaching the maximum effort, the subjects were verbally encouraged to continue the test for as long as possible (Midgley et al., 2018).

**TABLE 1:** Bruce test protocol description.

Stage	Duration (min)	Speed (km/h)	Grade (%)	≈MET (Ainsworth 1993)
Standing	3	0	0	2
1	6	2.7	10	5
2	9	4	12	7
3	12	5.4	14	10
4	15	6.7	16	13
5	18	8	18	16
6	21	8.8	20	18
7	24	9.6	22	20
8	27	10.4	24	22
9	30	11.2	24*	24

*Note: 24% was the maximum grade of the treadmill.

One MET (metabolic equivalent) equals an oxygen consumption of 3.5 ml/kg body weight/min, which is roughly equivalent to the basal metabolic rate in a stationary sitting position. The increase in oxygen consumption and energy expenditure can be expressed as manifolds of “MET.”

All tests were carried out in the same air-conditioned laboratory under standardized conditions (19°C–22°C ambient temperature, 99–102 kPa ambient air pressure, and 40%–50% relative ambient humidity) and were conducted in the same 3-h morning window to avoid possible circadian influences on the test (Atkinson and Reilly, 1996). All subjects were asked to avoid sleep deprivation the previous night, as well as to abstain from alcohol and drug consumption (except for oral contraceptives for the female subjects) and from caffeine the morning of the test. During the test, all subjects wore light sportswear of their choice (e.g., T-shirt and sweatpants) and wore the required measurement sensors (a face mask with turbine, HR monitor, and accelerometer watch), as well as a harness system to prevent injury from falling on the treadmill. Each subject was equipped with a wearable triaxial accelerometer on her/his non-dominant wrist (GENEActiv Original by Activinsights, UK). Sampling frequency of the acceleration measurement was 100 Hz. Recordings were stored in the onboard memory and downloaded offline after the experiment ended.

Trained and untrained subjects were selected from the participants of a previous work (Balcerek et al., 2020). The subjects were grouped based on their weekly training hours. Subjects who trained three times per week for a minimum of 1 h per session for at least 1 year were classified as “trained” (T), and subjects who did no or only occasional sports activity were classified as “untrained” (U). The max VO_2_ values have been obtained with the Bruce test. The reliability of the Bruce test in determining VO_2_ max has been demonstrated in many studies [see for example, Hall-López et al., (2015)].

### Algorithms and Outcome Variables

**FIGURE 1:**
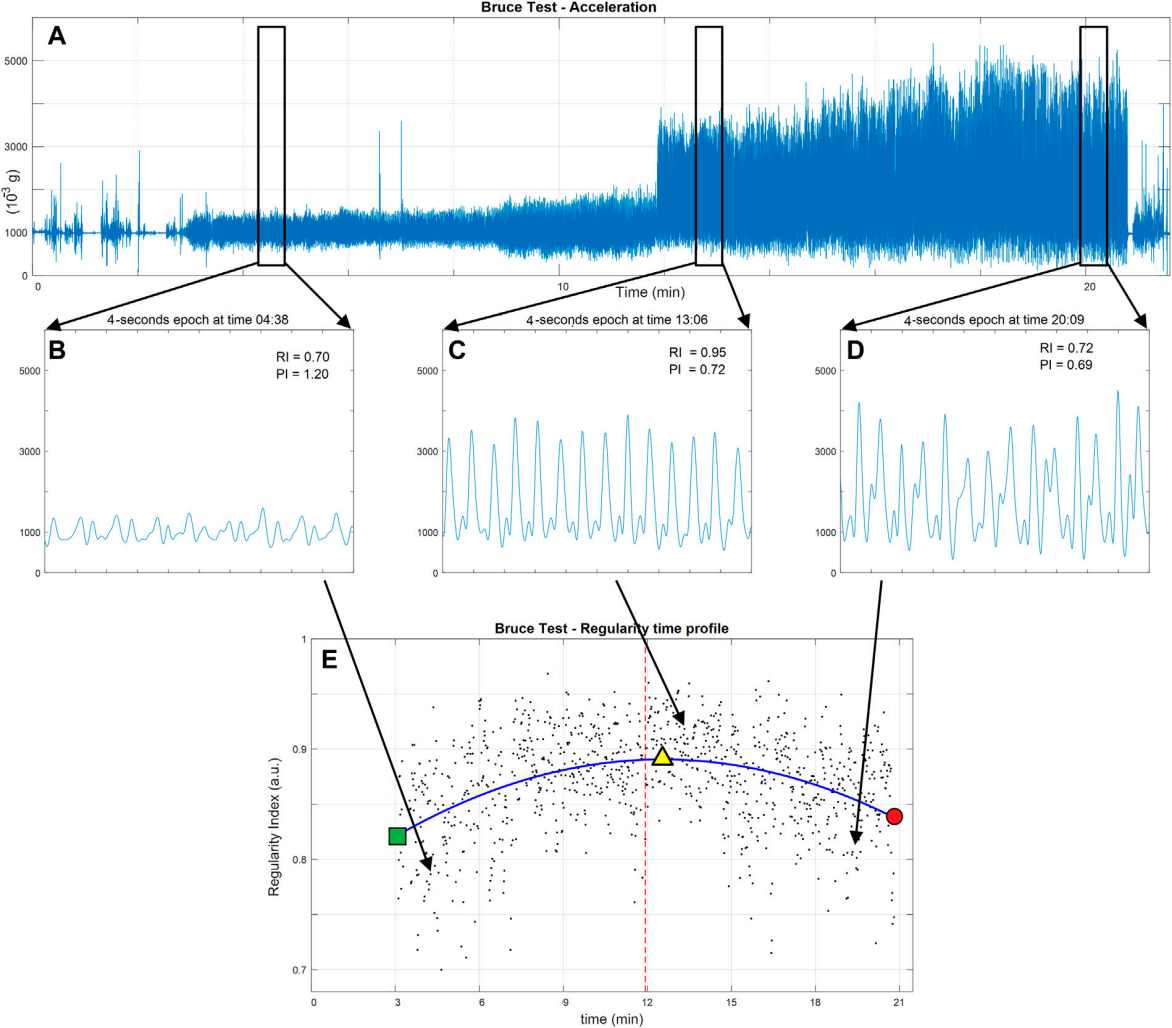
Data flow in the analysis of regularity of a Bruce test performance in a representative subject. **(A)** The norm of the acceleration as measured by a wrist triaxial accelerometer. **(B–D)** Zoom-in on the acceleration pattern in three representative 4-s epochs [**(B)** initial slow walking, **(C)** running following the walk–run transition, and **(D)** running at high-speed just before the test ends due to physical exhaustion). In the upper right-hand corner of each plot, the epoch’s regularity index (RI) is presented along with the duration of the locomotor cycle (PI). The lower panel **(E)** shows the scatter plot of all regularity indices computed for all epochs (black dots). The time pattern of regularity (blue line) is identified as the fitted second-order polynomial. The walk-to-run transition is presented as a red vertical line. The start of locomotion test is marked on the regularity pattern by the green square and its end by the red dot. The peak of regularity is marked by the yellow triangle. Time (in minutes) is in accordance with the Bruce test protocol.

The norm of triaxial acceleration recordings was filtered using a zero-lag fifth-order low-pass Butterworth filter with a cut-off frequency of 10 Hz (Figure 1A). This was done after having verified that the filter did not alter the information content of the raw signal. The filtered sequence was then analyzed in 4-s epochs extracted every second, thus partially overlapping (Figure 1B–D provides examples of this at different test stages). In each epoch, regularity and periodicity indices were computed according to an autocorrelation-based method reported in a previous work (Rabuffetti et al., 2019). The regularity index (RI) is the autocorrelation coefficient corresponding to the maximum peak value of the autocorrelation function with values ranging from a null value for a completely irregular acceleration pattern to a unitary value for an ideal periodic acceleration pattern. The periodicity index (PI) is the time (in seconds) of the maximum peak in autocorrelation, corresponding to the stride time of the considered locomotor act. The wrist position of the sensor, which lies away from the anatomical sagittal plane, did not allow for the identification of a secondary regularity peak that measures symmetry of movement.

The values of the RI in all epochs were characterized by pseudo-periodic behavior requiring the RI value to be above 0.70 (Rabuffetti et al., 2019). These values were plotted against their timing to produce the regularity pattern (Figure 1E). To verify the study hypotheses, we assumed that the following parametric second-order polynomial equation fits the curve:

$$RI\left(t\right)=at^{2}+bt+c$$

The equation was made to fit (in a least square sense) the above-defined time pattern of the RI. The fitted polynomial allows for the extraction of the following global indices:

• RI_start_—the initial value of the second-order polynomial fitted on the RI pattern (*Y*-coordinate of the green square point in Figure 1E)
• RI_peak_—the extreme value of the second-order polynomial fitted on the RI pattern (*Y*-coordinate of the yellow triangle point in Figure 1E)
• *T*_peak_—the timing of the RI_peak_ (*X*-coordinate of the yellow triangle point in Figure 1E)
• RI_end_—the final value of the second-order polynomial fitted on the RI pattern (*Y*-coordinate of the red dot in Figure 1E)
• *T*_end_—the timing of the RI_end_ (*X*-coordinate of the red dot in Figure 1E)
• Δ*T*_exhaustion_—computed as *T*_end_ − *T*_peak_
• RI_sharp_–the RI pattern sharpness (i.e., the second-order coefficient of the second-order polynomial fitted on the RI pattern). The sign of this index indicates in which direction the second-order polynomial opens (positive upwards and negative downwards).

Moreover, the time pattern of the PI (i.e., the time pattern of the stride duration) allows for the identification of the timing of the walk-to-run transition as the largest abrupt decrease in the periodicity profile (stride time profile). The following global indices relate to the walk-to-run transition:

• *T*_transition_—the timing of the walk-to-run transition (vertical dashed red line in Figure 1E)
• *T*_transition2peak_—computed as *T*_peak_ − *T*_transition_.

Time was measured in minutes, with the start of the Bruce test being the zero value. Regularity indices were non-dimensional and had values ranging up to the unity value (the null value marks the absence of any regularity; the unity value marks the occurrence of a perfect periodical pattern).

### Statistics

The balanced distribution across gender and training level of the experimental group was verified by a *χ* ^2^ test; differences in age and body mass index (BMI), associated with gender and training level, were verified by a Kruskall–Wallis analysis of variance.

It was important to verify whether the parabolas were congruent with the hypothesis stipulating an initial increase in regularity followed by a decrease in regularity. In order to do this, we did the following: 1) a strictly negative sign of RI_sharp_ was evaluated against a binomial distribution (i.e., the negative second-order coefficient of the polynomial that fits the individual regularity profiles was evaluated against binomial distribution) and 2) a strictly positive sign of Δ*T*_exhaustion_ (i.e., a non-null value characterizes a detectable exhaustion phase) was evaluated against a binomial distribution.

To verify whether differences between T and U subjects occurred and to quantify those differences, a non-parametric Wilcoxon test (*p* = 0.05, corrected for multiple tests according to Holm–Bonferroni) was performed for all computed global indices. The effect size was computed for the variables having a significant *p*-value by computing the Cliff’s delta (the sharper the differences between the groups, the closer to one the Cliff’s delta will be. No differences result in a null Cliff’s delta).

To verify whether physical characteristics are associated with indices related to the Bruce test and to regularity in particular, non-parametric Spearman rank correlation analyses (*p* = 0.05, corrected for multiple tests according to Holm–Bonferroni) were applied to matched series of subjects’ BMI values and performance indices.

## Results

Fifty subjects were enrolled in our study and were instructed to do a cardiopulmonary exercise test. Due to technical issues and data quality, as well as a few subjects dropping out, five subjects were excluded from the data analysis. The consolidated experimental group (*n* = 45) had a balanced distribution in terms of gender and training classification (*χ* ^2^ test, n. s.) and with no bias in age and BMI (Kruskall–Wallis test, n. s.).

As expected, the VO_2_ max values obtained from the incremental tests were significantly higher in T (56 mlO_2_ kg^−1^ min^−1^, range 44–81) vs U (47 mlO_2_ kg^−1^ min^−1^, range 35–57) subjects (*p* < 0.001 between groups) and remained higher considering the effects of sex (*p* < 0.001 T vs U women and *p* = 0.002 T vs U men).

Their test performances were efficaciously analyzed by the proposed method, and a second-order polynomial regression function consistently described the regularity data. Similarly, the periodicity profile, related to locomotor cadence, was consistent in its identification of the walk-to-run transition as the largest drop in stride duration.

**FIGURE 2:**
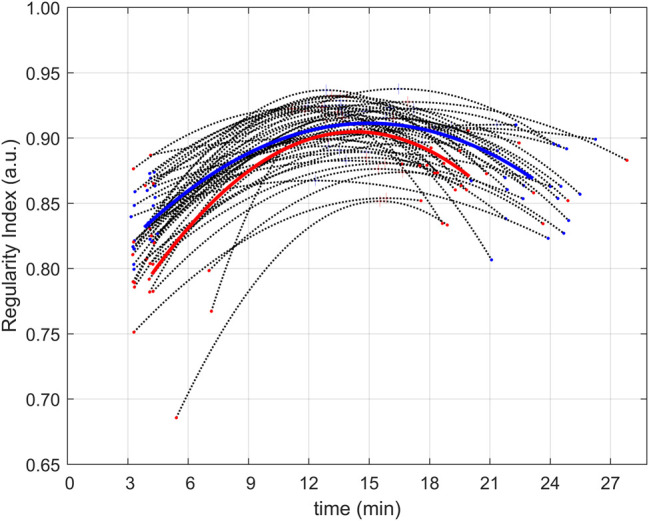
Regularity profiles of all subjects. Regularity profiles (computed as second-order polynomials fitting experimental data) for the 45 subjects (black dot lines with extremes and peaks, respectively, and dots and a plus (+) sign in red for untrained subjects and blue for trained ones). Average regularity profiles (in the form of second-order polynomials) are presented for the groups of untrained (red) and trained (blue) subjects.

A summary of the fitted second-order polynomials is presented in Figure 2.

All global indices were computed, and summary descriptors are reported in Table 2, where they are grouped by training level. The statistical comparisons of all indices between the T and U groups have been added to Table 2 as raw *p*-values (a hashtag marks significant differences in the *p*-value, corrected according to the Holm–Bonferroni method). Three indices showed significant differences between groups: in trained subjects, RI_start_ was about 4% higher, test duration *T*_end_ was about 19% longer, and duration of the final fatigue phase was about 32% longer when compared to untrained subjects. The largest effect size (0.71), quantified by the Cliff’s delta, was observed for *T*_end_, and the smallest effect size (0.52) was still relevant for RI_start_.

**TABLE 2:** Demographic and performance characteristics.

Param/index	Untrained (*N* = 23)					Trained (*N* = 22)					Raw *p*-value (Cliff’s delta)
	Min	Q1	Median	Q3	Max	Min	Q1	Median	Q3	Max	
**Age (years)**	19	22	25	31	40	22	24	28.5	32	37	0.2640
**BH (cm)**	157	172	178	183	195	151	170	174	182	190	0.4029
**BW (kg)**	50.5	61.5	71.8	78.3	95.0	47.0	65.8	71.8	75.4	90.0	0.9037
**BMI (kg/m** ^**2**^ **)**	18.7	20.9	22.9	23.9	25.8	17.9	21.4	22.9	24.9	26.4	0.3614
**RI**_**start**_	0.701	0.796	0.810	0.831	0.888	0.808	0.826	0.845	0.863	0.885	0.0016^#^ (0.52)
***T***_**transition**_ **(min)**	9.1	9.4	12.1	12.1	12.3	9.1	12.0	12.1	12.1	12.1	0.4748
***T***_**transition2peak**_ **(min)**	−2.1	1.1	2.5	3.6	6.4	−1.0	0.7	2.8	5.2	6.4	0.7332
***T***_**peak**_ **(min)**	10.0	12.5	13.8	15.3	16.4	11.1	12.6	13.4	16.1	18.4	0.5900
**RI**_**peak**_	0.852	0.881	0.909	0.921	0.931	0.873	0.897	0.915	0.925	0.937	0.2764
***T***_**end**_ **(min)**	15.6	18.2	18.6	21.3	27.1	18.9	21.0	23.0	24.1	25.4	0.0001^#^ (0.71)
**RI**_**end**_	0.833	0.858	0.873	0.882	0.907	0.810	0.850	0.867	0.891	0.906	0.5601
**RI**_**sharp**_ **(10** ^**−3**^ **)**	−2.46	−1.12	−0.80	−0.63	−0.27	−1.40	−0.71	−0.60	−0.39	−0.12	0.0104^§^
***T***_**exhaustion**_ **(min)**	1.4	3.9	6.5	7.2	11.4	2.9	7.3	8.6	9.7	11.8	0.0005^#^ (0.60)

^#^ *p* < 0.05, after correction, according to Holm–Bonferroni; ^§^not significant, after correction.

Statistical differences between trained (T) and untrained (U) groups in terms of anthropometric and performance indices. Min.–max. values, first and third quartiles (Q1 and Q3), and median values are reported. Effect size is reported for performance indices with a significant *p*-value, quantified by the Cliff’s delta.

As for the correlation between the performance indices and the individual BMI of the subjects, the only significant observation (Spearman test, rho = 0.496, raw *p*-value <0.001) was that of a linear positive relationship between RI_peak_ and BMI.

In addition to the *T*_transition_ values presented in the table, it is worth mentioning that the walk-to-run transition was a unique event for all included subjects (except for two subjects who switched from one strategy to another). This occurred around stage transitions, thus marking a change in locomotor strategy in response to changed external conditions (speed and slope increases). A minority of subjects (8 U and 5 T subjects) switched to running at the beginning of stage 4 (at about minute 9); all other subjects (15 U and 17 T subjects) switched at the beginning of stage 5 (at about minute 12).

Though the second-order polynomial fitting was not restricted to a sub-family of parabolas, the experimental data showed without exception that a downward parabola (strictly negative RI_sharp_) fits the regularity pattern and that the parabola apex was always included in the data duration. This implied that a non-null time duration (i.e., strictly positive Δ*T*_exhaustion_) characterized the regularity decrease at the end of the test. The shape of the curve does not include a single occurrence of a positive RI_sharp_ and not a single null Δ*T*_exhaustion_. Therefore, a binominal distribution test rejected the possibility that the regularity profile during a Bruce test is anything other than a phenomenon that increases at first and then decreases in the final stage.

## Discussion

In this paper, we evaluated the association between fatigue and locomotion regularity during the Bruce test and assessed whether training status may play a role in this relationship. Our results showed that locomotion regularity increases with speed and decreases with fatigue. Furthermore, our study shows that training status may affect such relationship throughout the whole exercise protocol.

Our results also confirm that the association between fatigue and locomotion regularity during the Bruce test, as well as the progression of fatigue effects throughout the exercise, can be successfully assessed by an autocorrelation method. Moreover, our hypothesis that gait variability and fitness level will affect locomotion regularity has been proven as we found that training affects performance regularity-related aspects. Indeed, for example, the T group showed a longer duration of the exhaustion phase characterized by a monotone decrease in regularity in comparison to the U group. The experiment confirmed that regularity [one of the primary features of efficient locomotion (Auvinet et al., 2002)] can be quantified using methods already applied to steady-state locomotion levels (Moe-Nilssen and Helbostad, 2004). This is also the case when complex and prolonged locomotor protocols such as the Bruce test are conducted. In fact, the Bruce test includes continuous simultaneous changes in both protocol features (i.e., slope and speed). These two factors impact and reflect an individual’s physical condition due to the emerging fatigue. In these applications, the expected modulation of the quantified time profile of regularity can be interpreted according to the variation in test features and of individual’s conditions.

We hypothesized that 1) initially, locomotor regularity would increase due to increased speed and that fatigue would be absent or would at most play a negligible role. 2) At higher speeds, only fatigue would reduce locomotion activity (due to fatigue playing a more dominant role as the Bruce test progresses).

The simplest mathematical model that implements such a hypothesized pattern is the second-order polynomial curve: the parabola. Parabolas that open downwards are the only types of parabolas with an identifiable peak value. This specific parabolic shape perfectly describes a smooth pattern of a variable and its derivative in a phenomenon where values increase in magnitude, reach a peak value, and then decrease. Such continuous modulation of regularity agrees with the observed progressive decreasing of foot clearance in prolonged locomotion overcoming obstacles (Heijnen et al., 2012). This model has been preferred over a two-piece-wise linear model due to the abrupt change in values at the walk-to-run transition, which would be more difficult to adapt in the observed physiological phenomena.

The chosen parabolic model proved well-suited to the patterns of regularity index obtained in the subjects who provided valid measurements: the fitted parabola complied, without exceptions, with the expected features: 1) a negative second-order coefficient and 2) a vertex inside the fitted section indicating a final phase of non-null duration characterized by decreasing regularity.

The regularity at the start of the locomotion test [RI_start_ (overall median 0.827, ranging from 0.701 to 0.888)] has values that are approximately 10% lower than published data concerning level walking at low speed (RI median 0.909, range 0.668–0.953) (Rabuffetti et al., 2019). Such small reduction in regularity, compared to the reference data, may be attributed to two factors: 1) slope—which is 10% in first stage of the Bruce test. While absent in the reference data, the incline is relevant in uphill locomotion (Ardigò et al., 2003). 2) Inferior gait speed (2.7 km/h) in the first stage of the Bruce test [especially when compared to the gait speed observed in reference data (3.6 km/h)].

The timing of the transition from walking to running (identified in the time profile of the periodicity index) occurred at the beginning of stage 5 for most subjects (32 out of 45 subjects, 8.0 km/h and 18% slope). In the minority of subjects, the transition from walking to running occurred at the beginning of stage 4 (13 out of 45 subjects, 6.7 km/h and 16% slope). This was in accordance with a previous study (Rotstein et al., 2005). Interestingly, the training level of subjects had no effect on the transition timing, whereas the role of training had been theoretically hypothesized to affect this (Beaupied et al., 2003).

The peak in the regularity profile is generally reached after the walk-to-run transition. The duration of this transition is quantified by *T*_transition2peak_. The median value of *T*_transition2peak_ is slightly less than the duration of one stage in the protocol (3 min) and may range up to about two stages in duration. On the contrary, only five out of 45 subjects reached the peak of regularity before the walk-to-run transition (resulting in a negative *T*_transition2peak_) with the anticipation being about 2 min at the maximum. In three other subjects, the anticipation was a matter of few seconds. Therefore, the onset of the decrement of regularity due to increasing effort and fatigue generally takes place when running after the walk-to-run transition. Interestingly, a review from Kung et al. (2018) reported that the preferred transition speed is generally slower than the theoretical energetically optimal transition speed, which happens to be approximately concurrent with the onset of the decrement of regularity.

The peak of regularity (overall median 0.912, ranging from 0.852 to 0.937) has values that are approximately 5% lower than published data concerning level running just above the transition speed (median 0.973, range 0.902–0.989) (Rabuffetti et al., 2019). Such a difference may be attributed to two factors: 1) the slope—which was not considered in the cited article but obviously plays a relevant role in uphill locomotion (Ardigò et al., 2003) and 2) cumulative fatigue—when the peak of regularity is reached during a Bruce test, the subject has already walked/run for 10 min and more, whereas the cited article (Rabuffetti et al., 2019) considered only 1-min bouts.

These findings support the interpretation of the timing of regularity peak as a marker of the onset of a final phase characterized by fatigue, ultimately leading to exhaustion. According to this interpretation, the duration of the fatigue phase (Δ*T*_exhaustion_) (i.e., a “final phase” during which regularity decreases) was about 7.26 min (median value, range 1.42–11.81 min). By the time the test ended, the regularity of locomotion (RI_end_) had dropped down to still relatively high values (median 0.869, range 0.810–0.907), indicating that exhaustion was reached without disrupting the locomotor patterns that still maintained a pseudo-periodic nature. This result confirms what was shown in previous studies (albeit with some methodological differences and different outcome variables). The main methodological difference was the continuous monitoring of the regularity index, whereas previous measurement sessions preceded and followed a fatiguing exercise (Schütte et al., 2015; Riazati et al., 2020). In one study, the outcome variables included the regularity (Schütte et al., 2015), while in another study, a space-state approach was adopted to quantify variability (Riazati et al., 2020).

The relatively short fatigue phase lasting only a few minutes may be due to the exponential increase in physical demand imposed by the continuously incremental physical demands of the Bruce test. Considering the features of the Bruce test (which is designed to reach the maximal effort level in a relatively short time), this was expected. Furthermore, it is well known that when effort intensity overrides the ventilatory threshold, the anaerobic energetic mechanism becomes more and more prevalent and induces metabolic acidosis within a short amount of time, which in turn interrupts the exercise.

The correlation analysis between the outcome indices and the subjects’ physical characteristics (quantified by BMI) revealed a moderate association (rho = 0.496, raw *p*-value <0.001) for only the RI_peak_, where individuals with higher BMIs were characterized by having a slightly higher peak in regularity. This partial association can be explained by two factors: 1) our subjects with higher BMI had more relevant muscular mass (none were overweight) and 2) larger inertial mass intrinsically resists more to status variation.

With regard to differences between T and U subjects, the test duration (*T*_end_) was significantly longer in T subjects (see Table 2), as would be expected in the Bruce test (Kang et al., 2001). Interestingly, the duration of the fatigue phase (Δ*T*_exhaustion_) was significantly longer in T subjects (see Table 2) as they were better at tolerating fatigue and were able to counteract the onset of metabolic acidosis. The significantly larger values of initial regularity RI_start_ in T subjects show how exercise training is associated with a more precise control of movements—particularly those related to locomotion. Such differences were confirmed by increased sharpness of the fitting parabolas (RI_sharp_) in U subjects (although this evidence was downgraded to a simple non-significant trend after correction for multiple tests).

As for regularity, the timing and magnitude of the peak in the regularity index (RI_peak_) were not significantly different between the T and U groups, confirming the absence of associations found in previous studies between gait variability and training level (Ciprandi et al., 2018). It is widely accepted that regular training (especially when starting in early childhood) causes improved neuro-muscular coordination of movement. That, in turn, is suggested to contribute to optimized energy handling during exercise, leading to enhanced performance. In contrast, our data strongly indicate that people with training experience do indeed show a higher regularity index at the beginning of the Bruce test (i.e., a significantly higher RI_start_ and a trend for lower RI_sharp_) when rates of respiration and circulation (oxygen delivery to working muscles) do not limit muscle work. However, no alteration in the efficiency of locomotion was observed at the peak of the regularity index (RI_peak_) or at the point of fatigue (RI_end_). This finding can be interpreted as a general ability of any healthy adult, irrespective of her/his training level, to perform efficient locomotion. We therefore suppose that neuro-muscular coordinated movement patterns do not contribute to the superior performance of T adults.

The limitations of the study primarily lie with the choice of the Bruce test. This is a classic test used in physiological experiments concerning metabolic and cardio-respiratory function (Bruce et al., 1963) that may appear unsuitable for biomechanical analysis. Indeed, the Bruce test combines two factors involving an ever-increasing physical demand: incremental increases in locomotion speed and increasing slope every 3 min. In this protocol, those two factors increasingly challenge the subject’s endurance in a relatively short time frame. The 3-min phase structure allows for a precise measurement of metabolic and cardio-respiratory performance. This combination may challenge biomechanical analysis given that there are two concurrent factors affecting locomotion patterns. However, the value and the justification of selecting the Bruce test rely exactly in the integration of regularity, and more in general of any biomechanical indices, in a concurrent continuous analysis of those physiological parameters, even considering the large number of studies involving this paradigm available insofar.

## Conclusion

The quantitative assessment of locomotor regularity in healthy adult subjects during a very demanding and escalating physical test provided some numerical indices related to performance and allowed for the identification of specific sub-phases. The presented experimental results showed the following:

1) Regularity increased in the first stage of the test (which was expected due to the increased locomotor speed); 2) the moment at which regularity reaches its peak value generally follows the walk-to-run transition; 3) from the moment of peak regularity onwards, fatigue generated by the test protocol escalation plays a major role in decreasing locomotion regularity; 4) the regularity profile can be described as a parabolic profile (second-order polynomial); and 5) the difference in test duration between U and T subjects is determined by a difference in the fatigue phase, characterized by decreased regularity, with the T group being able to prolong the final endurance phase.

Such detailed biomechanical insight, combined with the Bruce test’s cardio-respiratory and metabolic outcomes, can be integrated for a cross analysis and a comprehensive assessment of human physical performance. Moreover, these parameters are expected to influence functional assessment in studies involving subjects with limited physical abilities (e.g., individuals affected by mild to moderate neuromuscular deficits). The results of such studies could then provide more accurate monitoring and follow-up of therapeutic or rehabilitative interventions.

## Data Availability

The datasets for this study can be found in figshare at https:/doi.org/10.6084/m9.figshare.14778417.

## Footnotes

1
https://www.wma.net/policies-post/wma-declaration-of-helsinki-ethicalprinciples-for-medical-research-involving-human-subjects/
